# MetNet: A two-level approach to reconstructing and comparing metabolic networks

**DOI:** 10.1371/journal.pone.0246962

**Published:** 2021-02-12

**Authors:** Nicoletta Cocco, Mercè Llabrés, Mariana Reyes-Prieto, Marta Simeoni

**Affiliations:** 1 Dipartimento di Scienze Ambientali, Informatica e Statistica, Università Ca’ Foscari Venezia, Venice, Italy; 2 Mathematics and Computer Science Department, University of the Balearic Islands, Palma, Spain; 3 Evolutionary Systems Biology of Symbionts, Institute for Integrative Systems Biology (I 2 SysBio), Universitat de Valencia, Paterna, Valencia, Spain; 4 Sequencing and Bioinformatics Service, Foundation for the Promotion of Sanitary and Biomedical Research of the Valencia Region (FISABIO), València, Spain; 5 European Centre for Living Technology (ECLT), Venice, Italy; Institute of Evolutionary Biology, Pompeu Fabra University, SPAIN

## Abstract

Metabolic pathway comparison and interaction between different species can detect important information for drug engineering and medical science. In the literature, proposals for reconstructing and comparing metabolic networks present two main problems: network reconstruction requires usually human intervention to integrate information from different sources and, in metabolic comparison, the size of the networks leads to a challenging computational problem. We propose to automatically reconstruct a metabolic network on the basis of KEGG database information. Our proposal relies on a two-level representation of the huge metabolic network: the first level is graph-based and depicts pathways as nodes and relations between pathways as edges; the second level represents each metabolic pathway in terms of its reactions content. The two-level representation complies with the KEGG database, which decomposes the metabolism of all the different organisms into “reference” pathways in a standardised way. On the basis of this two-level representation, we introduce some similarity measures for both levels. They allow for both a local comparison, pathway by pathway, and a global comparison of the entire metabolism. We developed a tool, *MetNet*, that implements the proposed methodology. *MetNet* makes it possible to automatically reconstruct the metabolic network of two organisms selected in KEGG and to compare their two networks both quantitatively and visually. We validate our methodology by presenting some experiments performed with *MetNet*.

## Introduction

Metabolism is characterised by metabolic functions determining the structure and properties of cells in any organism. These functions interact with one another creating a complex network structure. While metabolism has traditionally been divided into metabolic pathways, subsystems of metabolism dealing with specific functions, it has become increasingly clear that metabolism operates as a highly integrated network [1, 2]. The research on the metabolomics field has considerably increased since the early 2000s, from the analysis of single pathways [3–6], to the comparative analysis of a set of pathways [7], together with the metabolic networks dynamics [8, 9]. All these studies are aimed to find out how the metabolism of different species has evolved in order to discern their associated metabolic functions (see for example [10, 11]), which are important for studying diseases and identifying pharmacological targets (as explored in [12, 13]).

Various approaches to metabolic network reconstruction, analysis and comparison can be found in literature, see [14–17] for surveys on different approaches and tools. Each approach chooses a representation of metabolic pathways that models the information of interest, proposes a similarity or a distance measure and possibly supplies a tool. The automation of the whole process is enabled by the knowledge stored in metabolic databases such as BioCyc [18], BioModels [19] and KEGG (Kyoto Encyclopedia of Genes and Genomes) [20–22]. However, obtaining the metabolism is a difficult task which generally requires human intervention, since data repositories are incomplete heterogeneous and incoherent. Furthermore, the comparison and visualisation of metabolic networks is challenging from a computational point of view due to the huge number of chemical reactions involved in metabolism.

Regarding the reconstruction and visualisation of metabolic networks, in [23] a technique to reconstruct and visualise a metabolic network, which can focus at different levels to master the network complexity, is provided. At the first level, the nodes of the hypergraph represent metabolic pathways and the hyperedges represent the relations between the pathways. Each hypernode is linked to other nodes at the second level of the data structure. Second level nodes represent enzymes, connected to each other by enzyme relations. Virtual edges connect identical compounds in different pathways to allow the user to do interactive operations, like collapse and expand over the hypergraph. This representation is used for a top-down display and for the visual comparison of metabolic networks in different organisms or in different databases. Data for metabolic network reconstruction are taken from the KEGG or Metacyc databases. Also, a method for automatic reconstruction of metabolic networks from the KEGG database is illustrated in [24]. For a selected organism, directed graphs representing enzyme relations are built from KEGG pathways. Then, guided by the information in the organism-specific KO hierarchy, a recursive union of the enzyme graphs is performed to obtain the whole metabolic network. In a similar way, in [25] the tool *AutoKEGGRec* is presented. This tool automatically reconstructs the metabolic network of a single organism or a list of organisms from the KEGG database, retrieving all their reactions and corresponding linked genes. The reactions and compounds metabolic network is then created. Another resource for the reconstruction of metabolic reactions from newly annotated genomes is the KBase Predictive Biology platform [26]. In this platform it is even possible to ensemble and annotate a genome, to ultimately construct its metabolic network.

To deal with the visualisation problem of huge metabolic networks, in [27] a new methodology is proposed that contracts all the reactions from a biconnected component of the metabolic network into a single node. As a result of this contraction, the metabolic network is converted into a simple structure, a *metabolic DAG* to easily visualise the network connectivity. As far as the comparison of metabolic networks or pathways goes, most tools compare two metabolic pathways by means of their networks alignment [28–31], while others define similarity measures based on their reactions similarity or the topological properties of the networks [32–34]. However, they do not provide a graphical visualisation of the obtained results on metabolic networks comparison.

To fill this gap, we propose a new approach and a corresponding tool for the automatic reconstruction, comparison and visualization of metabolic networks based on KEGG data. We rely on KEGG as a source of metabolic data, because it is explicitly designed to present data in a standardised way. KEGG decomposes a metabolic network into modules, called *reference pathways*, each one of which is associated with a specific metabolic function. Since metabolic pathways are quite preserved among organisms, KEGG associates a unique reference pathway to each function in different organisms, which corresponds to the union of the corresponding pathways. For example, the reference pathway for *Glycolysis* is a graph representing the relations among reactions and metabolites in the glycolysis metabolism of all the organisms in the database. This decomposition into modules is not a partition of the metabolic network since each reference pathway can share reactions and metabolites with the others. Moreover, the KEGG database also presents an API to map genes to pathways and use multiple colors for the same node that enables highlighting unique enzymes or shared reactions between organisms of interest [35]. KEGG also provides a global metabolic map that allows for a bird eye view of the whole metabolism: the reference global map shows the various pathways in different colors, suggesting the idea of a two-level view of the metabolism.

Our approach to the automatic reconstruction and comparison of metabolic networks in different organisms overcomes the aforementioned computational problems by exploiting the information in KEGG and its standardised modularisation of metabolism. This is achieved by representing a metabolic network in two distinct levels: the *structural* level and the *functional* level. The higher structural level shows the overall structure of the metabolic network in terms of KEGG pathways and connections among pathways. Such connections are identified by the non-ubiquitous molecular compounds they share. The lower functional level represents the functional role of each pathway in the metabolic network in terms of its basic components, the reactions.

We developed a tool, *MetNet*, that implements our proposal. It allows the user to choose any pair of organisms within the KEGG database, to automatically reconstruct their metabolisms in the two-level representation, and to compare them in pairs. In order to perform the comparison, some similarity measures have been defined. The comparison method based on the two-level representation makes it possible to visualise the network structures and explore their similarities and differences, and to compute similarity indexes, associated both to the entire metabolism and to specific metabolic functions.

We validate the proposed methodology by presenting some experiments performed with *MetNet*. The first experiment is a pairwise comparison of two organisms that allows us to present the functionalities of the tool and to highlight the advantages of the two-level representation of the metabolism and the possibility of exploring the comparison results both quantitatively and visually. Two further experiments show an extended usage of *MetNet* as a tool to compare a set of organisms with the aim of unveiling useful structure and functional information about their metabolism, and to explore whether the groups suggested by our similarity indexes agree with those established by well known evolutionary relationships.

## Materials and methods

**Fig 1:**
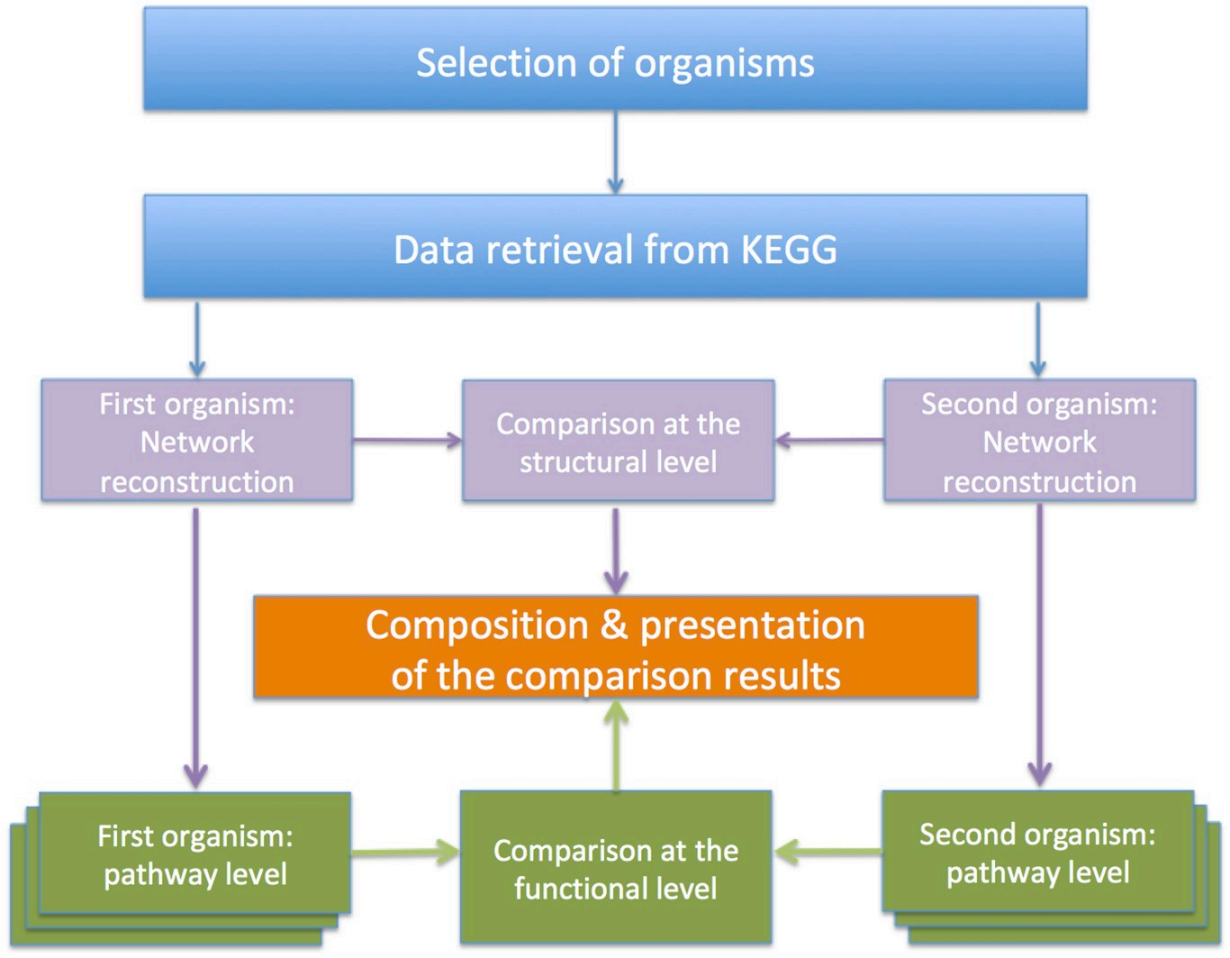
Pairwise metabolism comparison of different organisms: Overall view of the proposed methodology.

This section illustrates our methodology for automatically reconstructing metabolisms from KEGG metabolic data and for comparing metabolisms of different organisms. The overall view of the proposed reconstruction and comparison methodology is described in Fig 1. The first step consists of selecting two organisms to analyse. Subsequently, their metabolic data are retrieved from KEGG and the corresponding networks of metabolic functions are built. We propose a metabolism comparison method based on a two-level representation of metabolic networks: a *structural* level representing the metabolic network topology and a *functional* level representing the metabolic functions of each pathway. The approach is supported by similarity indexes for the comparisons at both levels. At the end, the comparison results are composed and presented to offer a comprehensive view of the similarities/differences of the two organisms.

### Metabolism representation and reconstruction from KEGG data

The first step in automatically reconstructing the metabolic network of a specific organism is automatic data retrieval. Our metabolism reconstruction method is based on the KEGG database. At present there are 542 Eukaryotes and 6397 Prokaryotes, divided into 6059 Bacteria and 338 Archea, within the KEGG database.

Since metabolic pathways are quite preserved among organisms, KEGG associates to each metabolic function a unique *reference pathway* which corresponds to the union of the corresponding pathways in all the organisms included in the database. A pathway of a specific organism can be obtained from the corresponding reference pathway. This standardised and modular representation of pathways plays an important role in our methodology to avoid incoherence in metabolism comparison.

**Fig 2:**
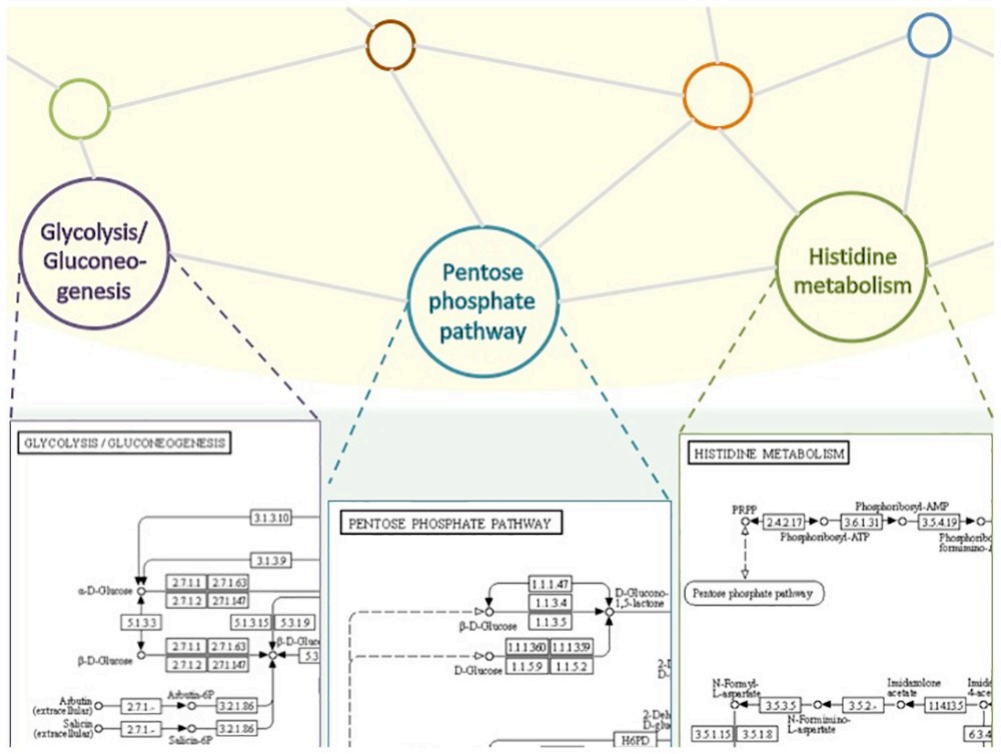
A two-level view of the metabolism: The *Structural level* (yellow background) and the *Functional level* (green background).

We view a metabolic network as a network of chemical reactions. Our reconstruction method represents such a network in two distinct levels: a higher *structural* level and a lower *functional* one, as illustrated in Fig 2.

At the structural level the metabolic network of a given organism is represented by a graph whose topology reflects the metabolic pathways stored in KEGG for that organism and their interconnections. Each node in the graph identifies a pathway of the given organism and an edge between two nodes states that the two corresponding pathways share one or more compounds (ubiquitous compounds such as H_2_O, phosphate, ATP and ADP are not considered). Note that a shared compound *C* in KEGG may represent different situations:

- *C* is produced by one pathway and consumed by the other;
- *C* is a compound used in the shared part of two overlapping pathways;
- *C* is a compound present in both pathways, even though they express unrelated functions or work in different environments or conditions.

The description at the higher level is very basic and a more concrete representation of all pathways is needed. This is achieved at the functional level, where the specific functions carried out by each metabolic pathway are taken into account by considering the chemical reactions that compose them. In particular, the concrete functions of each pathway are represented through the set of their chemical reactions.

The metabolic activity in KEGG is divided into the various categories shown in Table 1. Each category is then composed of various metabolic pathways. In order to consider the whole metabolic networks, all categories and all the corresponding pathways should be contemplated.

**Table 1:** List of KEGG metabolic categories: Each one is composed by many metabolic pathways.

Metabolic category
Carbohydrate metabolism
Energy metabolism
Lipid metabolism
Nucleotide metabolism
Amino-acid metabolism
Metabolism of other amino-acids
Glycan biosynthesis and metabolism
Metabolism of cofac tors and vitamines
Metabolism of Terpenoids and polyketides
Biosynthesis of other secondary metabolites
Xenobiotics biodegradation and metabolism

KEGG supplies two related representations for each pathway in its repository: a graphical representation (*pathway map*), showing the network of chemical reactions composing the pathway, and a textual one written in an XML format, a *KGML file*, where KGML stands for *KEGG Markup Language*. Such a file contains the information represented in the corresponding map. To automatically reconstruct the metabolism of a specific organism, it is necessary to download the KGML files of each pathway of the organism through the public KEGG’s APIs, and to parse each KGML file to extract the relevant information for our representation, which are the compounds and the reactions of each pathway.

If a pathway is not present in an organism, because the organism does not need it for its metabolism, the corresponding KGML file does not exist. KGML files only contain reactions information for pathways that include a gene/protein or a chemical network, we call them *reaction pathways*. If a reaction pathway is present in the metabolism of an organism, its corresponding KGML file exists and contains one or more chemical reactions.

The pathways based on physical mechanisms, i.e. membrane pathways, do not contain information on reactions and are represented only as images in KEGG. Their KGML files exist but they do not contain any chemical reactions. Since we view the metabolism as a network of chemical reactions, they are represented in our network reconstruction as nodes at the structural level and as empty reaction sets at the functional level.

KGML files also contain information about how each pathway is linked to others, the so called *maplinks*. As maplinks are intended mainly for visual comprehensibility and are not reliable and complete, we decided not to use such information in constructing the structural graph.

Choosing the KEGG database as the unique source of metabolic data allows us to automatise network reconstruction and to benefit of KEGG’s modularization of metabolism into standardised functions therein avoiding incoherence in metabolism comparison. Clearly our automatic approach strictly depends on the data representation and on the knowledge available in KEGG, data incompleteness or inconsistency would reflect negatively on our comparison method. We can, however, count on the fact that KEGG is a widely known resource, constantly updated by its staff on the basis of new knowledge.

### Similarity indexes for metabolism comparison

After reconstructing the metabolisms of different organisms, we can study them by comparison. We define some similarity indexes for the comparison, associated to the two-level representation adopted in our methodology. Let us consider them bottom-up, a summary is presented in Table 2 for local similarity indexes and Table 3 for global similarity indexes.

**Table 2:** Summary of local similarity indexes.

Local Pathway Indexes
$SimP_{i}=\{\begin{array}{ll} 0 & \text{if}\;P_{i}\;\text{is}\;\text{present}\;\text{in}\;\text{one}\;\text{and}\;\text{only}\;\text{one}\;\text{organism}\;(O\;\text{or}\;O^{\prime })\\ 1 & \text{if}\;P_{i}\;\text{is}\;\text{a}\;\text{physical}\;\text{pathway}\;\text{present}\;\text{in}\;\text{both}\;O\;\text{and}\;O^{\prime }\;(\text{no}\;\text{reactions}\;\text{to}\;\text{compare})\\ \frac{\left|R_{i}\cap R_{i}^{\prime }\right|}{\left|R_{i}\cup R_{i}^{\prime }\right|} & \text{if}\;P_{i}\;\text{is}\;\text{a}\;\text{reaction}\;\text{pathway}\;\text{present}\;\text{in}\;\text{both}\;O\;\text{and}\;O^{\prime } \end{array}$
$SimS_{i}=\{\begin{array}{ll} 0 & \text{if}\;P_{i}\;\text{is}\;\text{present}\;\text{in}\;\text{one}\;\text{and}\;\text{only}\;\text{one}\;\text{organism}\;(O\;\text{or}\;O^{\prime })\\ 1 & \text{if}\;P_{i}\;\text{is}\;\text{isolated}\;\text{in}\;\text{both}\;O\;\text{and}\;O^{\prime }\\ \frac{1}{1+deg(P_{i})} & \text{if}\;P_{i}\;\text{is}\;\text{isolated}\;\text{in}\;O\;\text{and}\;\text{connected}\;\text{in}\;O^{\prime }\;\text{or}\;\text{vice}\;\text{versa}\\ \frac{\left|E_{i}\cap E_{i}^{\prime }\right|}{\left|E_{i}\cup E_{i}^{\prime }\right|} & \text{if}\;P_{i}\;\text{is}\;\text{connected}\;\text{in}\;\text{both}\;O\;\text{and}\;O^{\prime } \end{array}$

**Table 3:** Summary of global similarity indexes.

Global Indexes on metabolism	
$Psim=\frac{\sum_{i=1}^{n}SimP_{i}}{n}$	pathway similarity
$Psim_{W}=\frac{\sum_{i=1}^{n}SimP_{i}*\left|R_{i}\cup R_{i}^{{}^{\prime }}\right|}{\sum_{i=1}^{n}\left|R_{i}\cup R_{i}^{{}^{\prime }}\right|}$	weighted pathway similarity
$Ssim=\frac{\sum_{i=1}^{n}SimS_{i}}{n}$	structure similarity
$CSim=\frac{\sum_{i=1}^{n}SimS_{i}*SimP_{i}}{n}$	combined similarity

At the functional level we compare the same metabolic function between two different organisms. In our methodology this means comparing the same metabolic pathway from two different organisms, i.e. two pathways corresponding to the same reference pathway in KEGG. Let us consider two different organisms *O* and *O*′ and the i-th KEGG reference pathway *P*_*i*_. The comparison naturally relies on common reactions in the pathways corresponding to *P*_*i*_ in the two organisms. We adopt the simplest representation for a pathway, that is, we choose to represent it either as a set or as a multi-set of reactions. Then, our similarity index for a metabolic function, indicated with *SimP*_*i*_ and called *P*_*i*_ *pathway similarity index*, is based on the Jaccard index. We distinguish different cases to take into account the pathways based on physical mechanisms. The cases are listed in the same order as in Table 2.

**Case 1**. A pathway corresponding to *P*_*i*_ is present in one and only one of the two organisms. *SimP*_*i*_ is set to 0, the minimal similarity.
**Case 2**. A pathway corresponding to *P*_*i*_ is present in both organisms, but the corresponding KGML file does not contain any reaction information, i.e. *P*_*i*_ deals with physical instead of chemical transformations. *SimP*_*i*_ is set to 1, the maximal similarity. In this case a detailed comparison cannot be made, the index just considers that the function is present in the metabolism of both organisms.
**Case 3**. A pathway corresponding to *P*_*i*_ is present in both organisms and contains the reactions to be compared (reaction pathway). Let *R*_*i*_ and $R_{i}^{{}^{\prime }}$ be the sets (multi-sets) of the reactions of the pathway corresponding to *P*_*i*_ in *O* and in *O*′ respectively, then $SimP_{i}=\frac{|R_{i}\cap R_{i}^{{}^{\prime }}|}{|R_{i}\cup R_{i}^{{}^{\prime }}|},$ where $|R_{i}\cap R_{i}^{{}^{\prime }}|$ represents the number of common reactions and $|R_{i}\cup R_{i}^{{}^{\prime }}|$ represents the number of all the reactions in the two pathways. | | indicates the cardinality and ∪, ∩ the union and the intersection defined either on sets or on multi-sets, depending on the chosen pathway representation.

Note that *SimP*_*i*_ = 1 either when a physical pathway is present in both organisms or when a reaction pathway is present in both organisms and their set (multi-set) of reactions is the same. Indeed, in both cases, the metabolic pathways of the two organisms coincide in our representation.

To compare the complete metabolism of *O* and *O*′ at the functional level, two distinct pathway similarity measures are defined based on *SimP*_*i*_. The *pathway similarity index* is the arithmetic mean of all the pathways similarities:

$$Psim=\frac{\sum_{i=1}^{n}SimP_{i}}{n}$$

where *n* is the total number of KEGG pathways present in *O* or in *O*′. The *weighted pathway similarity index* is the weighted mean of the pathways similarities wrt. the number of reactions:

$$Psim_{W}=\frac{\sum_{i=1}^{n}SimP_{i}*\left|R_{i}\cup R_{i}^{{}^{\prime }}\right|}{\sum_{i=1}^{n}\left|R_{i}\cup R_{i}^{{}^{\prime }}\right|}$$

where *n* is the total number of pathways present in *O* or in *O*′. This second measure assigns a lower weight to the similarities of “smaller” reaction pathways. Both pathway similarity indexes, by definition, assume values in [0, 1]. Note that the pathway similarity index *Psim* considers all pathways independently of their reaction content, while the weighted similarity index *Psim*_*W*_ considers only reaction pathways, therefore ignoring pathways dealing with physical transformations.

At the structural level the metabolic network of an organism is represented as a graph. Let *G* = (*V*, *E*) and *G*′ = (*V*′, *E*′) be the graphs representing the metabolic networks of two organisms *O* and *O*′, respectively, and let *P*_*i*_ be a reference pathway corresponding to a node in *G* or *G*′ (or in both). The *P*_*i*_ *structure similarity index*, *SimS*_*i*_, is defined as follows (the various cases are listed in the same order as in Table 2):

**Case 1**. if one and only one graph has the node corresponding to *P*_*i*_, i.e. the metabolic pathway is present only in one organism, *SimS*_*i*_ is set to 0.
**Case 2**. if both *G* and *G*′ have the node corresponding to *P*_*i*_ and in both graphs the node is isolated, namely *P*_*i*_ does not share any compound with other pathways, *SimS*_*i*_ is set to 1.
**Case 3**. if both *G* and *G*′ have the node corresponding to *P*_*i*_, in one graph the node is isolated and in the other graph it is connected with degree *k* > 0 (i.e. *k* connections with other pathways), *SimS*_*i*_ is set to $\frac{1}{1+k}$.
**Case 4**. if both *G* and *G*′ have the node corresponding to *P*_*i*_ and in both graphs the node is connected, $SimS_{i}=\frac{|E_{i}\cap E_{i}^{{}^{\prime }}|}{|E_{i}\cup E_{i}^{{}^{\prime }}|}$, where *E*_*i*_, $E_{i}^{{}^{\prime }}$ are the sets of edges incident to the node in *G* and *G*′, respectively.

To compare the overall metabolism of *O* and *O*′ at the structural level, we consider the arithmetic mean of the structure similarities of all pathways. Thus, the *structure similarity index* is defined by:

$$Ssim=\frac{\sum_{1=1}^{n}SimS_{i}}{n}$$

where *n* is the total number of pathways present in *O* or in *O*′, that is |*V* ∪ *V*′|.

In our two-level representation, a global index is defined for comparing metabolic networks of two organisms, that is, taking into account their similarity at both levels. The *combined similarity index* is:

$$Csim=\frac{\sum_{i=1}^{n}SimS_{i}*SimP_{i}}{n}$$

where *n* is the number of KEGG pathways in the two organisms, |*V* ∪ *V*′|. By definition, the index assumes values in [0, 1].

Note that, when pathways based on physical mechanisms are present in the compared organisms *O* and in *O*′, the combined similarity index will stress their similarity/difference since it is set to 1/0. Note moreover that both *SimS*_*i*_ and *SimP*_*i*_ have values in [0, 1], hence the combined similarity index will amplify the differences between metabolisms.

## Results and discussion

In this section we present *MetNet*, a tool that implements the proposed methodology and allows us to validate our approach. First we illustrate the tool’s functionalities and then we report three experiments performed with *MetNet*. The first experiment compares the metabolism of two selected organisms with the aim of highlighting their relationships. Two further experiments compare groups of organisms, with the aim of exploring whether the groups suggested by our similarity indexes agree with those established by well known evolutionary relationships.

### *MetNet*: A tool for metabolic network comparison

*MetNet* is a Java tool that applies our reconstruction and comparison methods, the main steps of which are depicted in Fig 1. In particular, *MetNet* allows the user to select two organisms, to automatically retrieve their metabolic pathway data from KEGG using the public KEGG APIs, to reconstruct the metabolic network in our two-level representation, and to perform both a quantitative and a visual pairwise comparison of such networks. Being a Java application, *MetNet* is portable: it can run in any environment in which the Java Runtime Environment (JRE) is installed. Graph visualization is performed using the GraphStream library [36].

*MetNet* has been designed to be used in two different modalities:

- as **an interactive application** with a user friendly graphical interface which allows for the comparison and visualization of the metabolism of two organisms;
- as **a command line tool** that can be used in a broader context, e.g. as part of a computational pipeline involving different tools, or to compare a group of organisms instead of just a pair with an ad-hoc shell script.

Although the aim of the proposed methodology is to compare the entire metabolic network of different organisms, the list of metabolic pathways to be considered for the comparison can be specified through a configuration file, thus supplying the user with a flexible way to choose the metabolic aspects of interest to be considered for the comparison in each experiment.

The structural representation of the metabolic network of an organism is implemented by a squared adjacency matrix *m* whose rows and columns represent all the KEGG reference pathways considered for the comparison (i.e. the ones listed in the configuration file) and whose matrix entries represent the connections between pathways of that organism. In particular, each entry *m*[*i*, *j*] stores the number of common compounds between pathways *P*_*i*_ and *P*_*j*_. Such information is clearly symmetric, hence *m*[*i*, *j*] = *m*[*j*, *i*]. Entries of the main diagonal are instead used to store summary information about pathways: *m*[*i*, *i*] = −1 indicates that pathway *P*_*i*_ is not present in the metabolism of the organism; *m*[*i*, *i*] = 0 indicates that *P*_*i*_ is present in the metabolism but isolated wrt. the other pathways; *m*[*i*, *i*] = *k* > 0 means that *P*_*i*_ has *k* connections with other pathways in total.

By taking into account all the KEGG reference pathways considered in the comparison, we standardise the representation as matrices of metabolic networks and greatly simplify their comparison, since each position in the different matrices corresponds to the same pathway.

We present here the tool functionalities with the help of some images related to the first experiment performed with *MetNet* and illustrated later in this section: the pairwise comparison of the organisms *Acyrthosiphon pisum* (KEGG code api) and *Buchnera aphidicola 5A* (KEGG code bap).

**Fig 3:**
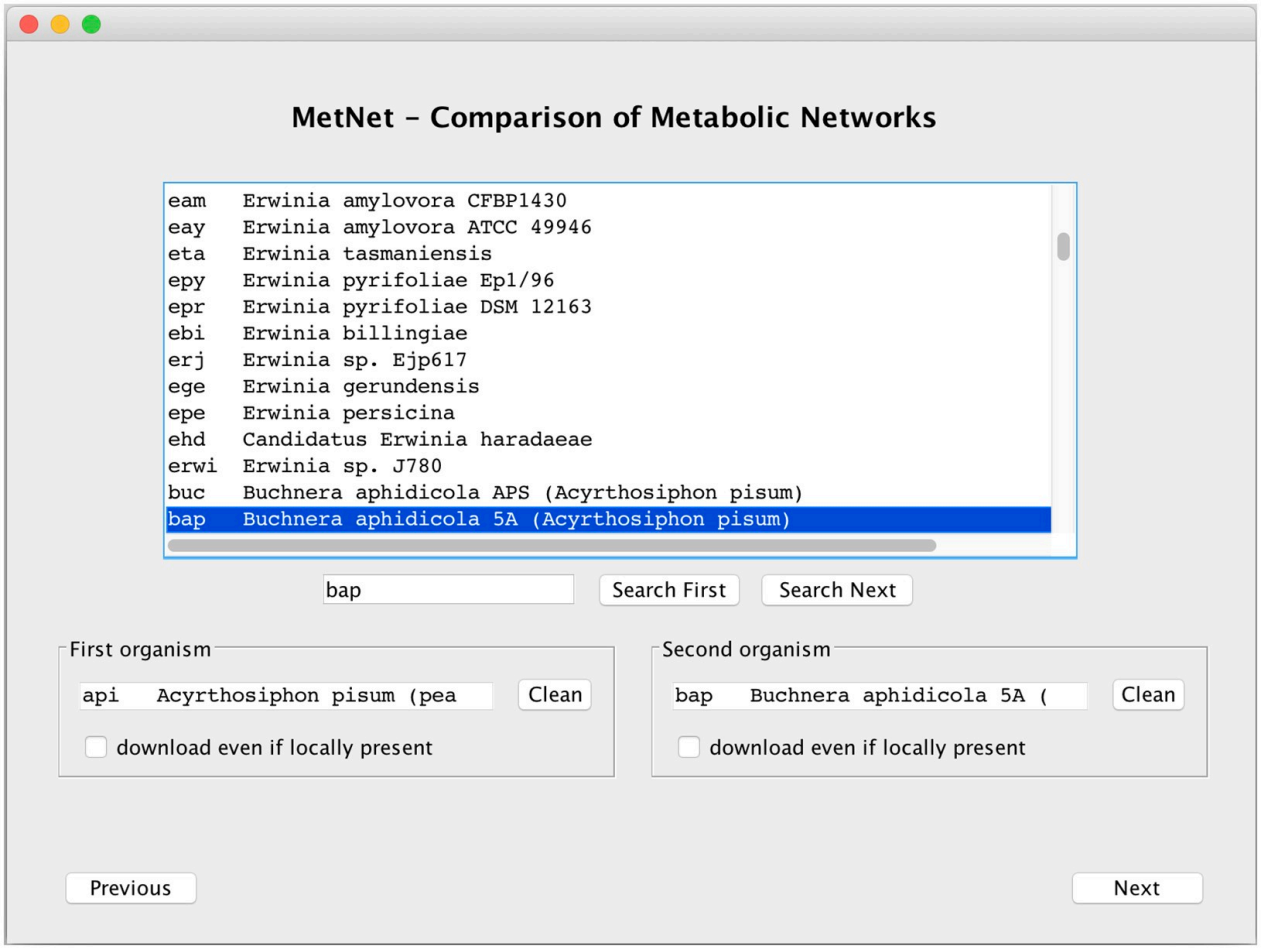
Organisms selection window: Example for api and bap.

When starting the application, the initial view allows the user to download the latest list of KEGG organisms: if the download is requested, the local list is substituted with the new one. The user is then driven to the organisms selection window, see Fig 3. The list of KEGG organisms is displayed in the upper part of the window: the user can search or scroll through the list and select the two organisms for comparison by double-clicking their rows. Once the two organisms have been selected, the “Next” button drives the user to the comparison window. If the two organisms are not locally present or the user requires a new version of their files, the downloading procedure of the KGML files is performed automatically before moving to the comparison window.

**Fig 4:**
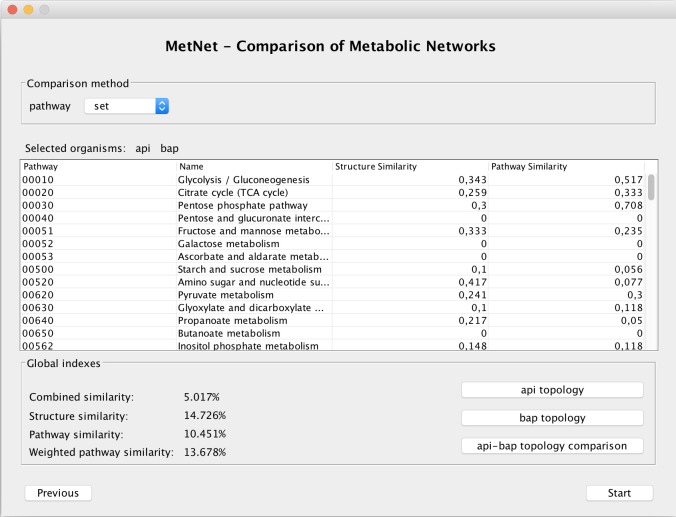
Comparison results for api and bap.

The comparison window allows the user to select the comparison method for the functional level (i.e. set or multi-set of reactions) and to start the comparison. The result of the comparison is shown in Fig 4. *MetNet* uses multithreading where possible: the workload is divided into different tasks that can be executed in parallel to increase performance. The computation requires an execution time that depends on the complexity of the networks. The average execution time is around one minute on a MacBook PRO with 16GB central memory.

Note in Fig 4 that the pathway comparison is displayed as a table of similarity values. The table has four columns that show, for the two selected organisms: the KEGG pathways ids, *i*, their names, *P*_*i*_, the similarity values computed for the local structure *SimS*_*i*_ and the pathway similarity values *SimP*_*i*_. Below the table, the global similarity indices, *Psim* and *Psim*_*W*_ for the functional level, *SimS* for the structural level and the combined similarity index *CSim*, are shown as well. All the local and global similarity results are also automatically saved as an Excel file stored in the *MetNet*’s main folder.

**Fig 5:**
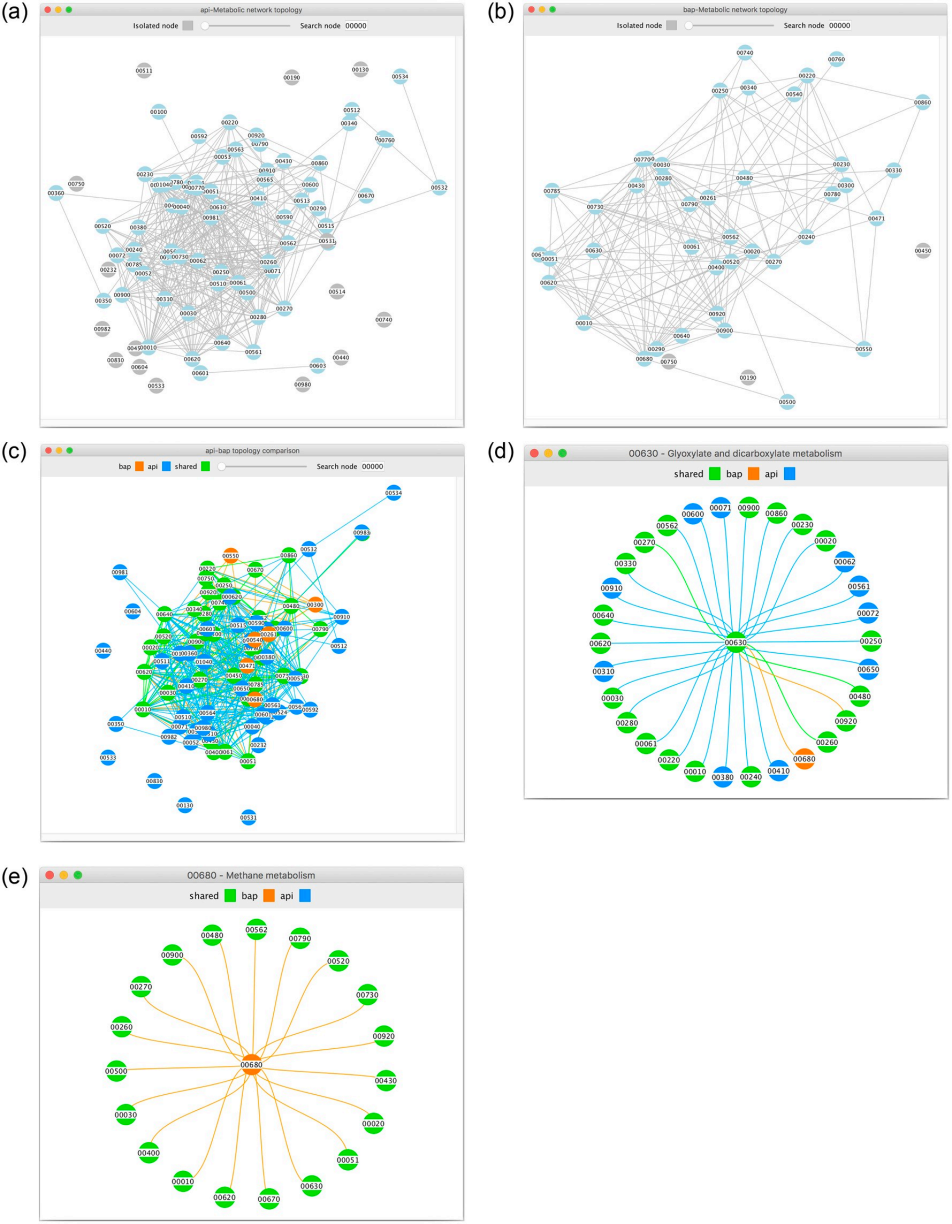
Visual comparison of api and bap at the structural level: **(a)** api metabolic network; **(b)** bap metabolic network; **(c)** api-bap topology comparison; **(d)** *Glyoxylate & dicarboxylate metabolism* pathway connections; **(e)** *Methane metabolism* pathway connections.

*MetNet* also offers the possibility of visualising the metabolic network topology of the two organisms separately through the corresponding buttons on the main window. Fig 5(a) and 5(b) show the api and bap metabolic networks, respectively. We recall that nodes in the graphs are the pathways and edges are the connections between pathways due to shared compounds. Each node shows its corresponding KEGG pathway id as a short label, but also its name and degree appears as a tooltip when the user hovers over the node with the cursor. The two graphs can also be compared visually through the topology comparison button that displays the shared and unshared parts of the network with different colors allowing also to distinguish the organism to which the unshared parts belong, as reported in Fig 5(c). Furthermore, each visualised graph can be inspected in three different ways: first, it is possible to search for a particular pathway, which will be highlighted if present in the graph. Second, by double clicking on a node, the node itself and its neighbors will appear in a separate window, so that the user can better inspect how other pathways are connected with the specific node of interest. An example is shown in Fig 5(d) and 5(e). Third, the visualized graph can be zoomed in to better focus on a specific part of the graph itself. This latter feature is particularly useful for large graphs.

### Experiments

We now proceed by showing some experiments performed with *MetNet*. When more than two organisms are involved in the comparison, we use *MetNet* as a command line tool and create a specific shell to include all the pairwise comparisons. Once all comparisons are completed, we make use of the excel files produced by *MetNet* to build the similarity matrices of all the obtained global indexes. Such matrices can be used to perform cluster analysis and check whether the organisms under exam are grouped according to well-known evolutionary relationships.

#### First experiment: A symbiont-host relationship

In this experiment we perform the pairwise comparison of an insect, *Acyrthosiphon pisum* or pea aphid (KEGG code api), and *Buchnera aphidicola 5A* (KEGG code bap), a proteobacteria that is the primary endosymbiont of A. pisum [37]. We are interested in exploring the relationships between their metabolisms through *MetNet*.

The results of their comparison is shown in Fig 4. We can observe that the two organisms are largely different for all indexes both at the structural and functional levels, as their global similarity indexes values range from 5.02% to 14.73%.

To understand this result, we must focus on the biology of these two organisms. They live together in symbiosis, the bacteria *Buchnera* is a symbionelle of the insect *A. pisum* (i.e., lives in specialised cells of the host’s body called bacteriomes, where its entire life cycle is developed) [38]. This type of association is commonly observed in nature, specifically in insects where organisms have evolved in such a manner that symbionelles basically work for their host. Since aphids survive on a very nutrient-poor diet (eating plant sap), the endosymbiotic bacteria they possess provide them with essential amino acids and nutrients in exchange for a rich and stable environment in which to live [39, 40]. Therefore, the result of low ranges of global similarity of their metabolisms is expected because they possess complementary metabolisms for the host to survive in it’s given environment.

The visual inspection of the api and bap networks topologies in Fig 5(a) and 5(b) confirms that the two networks are very different: api is an insect with a genome of 464 Mb that has a complex metabolic network [41], while bap is a bacteria with a reduced genome of 640 Kb [42] and has a much simpler network.

Moreover, Fig 5(d) shows the connections of the *Glyoxylate and dicarboxylate metabolism* pathway, and Fig 5(e) the relation between the *Methane metabolism* of bap and api. Both of these pathways were found to be very important pathways for the production of amino acids inside the bacteriocytes of api [43]. As the authors in [43] conclude, it is evident that these organisms are completely dependent on each other based on their distinctive metabolic complementation. Furthermore, these are only two examples of many recurrent metabolic complementations found in the association between insects and their endosymbiotic bacteria, as well as many other systems where bacteria are involved [44, 45]. Note that *MetNet* is able to clearly highlight this complementation in an easy and visual way.

#### Second experiment: Yeasts and Molds

For this experiment we selected eight organisms among Fungi, four Yeasts (*sce, zro, tpf, cal*) and four Molds (*fgr, tre, afm, abp*) that are listed in Table 4. The goal is to test the ability of our similarity indexes to discriminate between very similar organisms.

**Table 4:** Yeasts and Molds considered in the second experiment.

Code	Organism	Kingdom	Taxonomic group
*sce*	*Saccharomyces cerevisiae* (budding yeast)	Fungi	Saccharomycetes
*zro*	*Zygosaccharomyces rouxii*	Fungi	Saccharomycetes
*tpf*	*Tetrapisispora phaffii*	Fungi	Saccharomycetes
*cal*	*Candida albicans*	Fungi	Saccharomycetes
*fgr*	*Fusarium graminearum*	Fungi	Sordariomycetes
*tre*	*Trichoderma reesei*	Fungi	Sordariomycetes
*afm*	*Aspergillus fumigatus*	Fungi	Eurotiomycetes
*abp*	*Agaricus bisporus var. burnettii JB137-S8*	Fungi	Basidiomycetes

**Fig 6:**
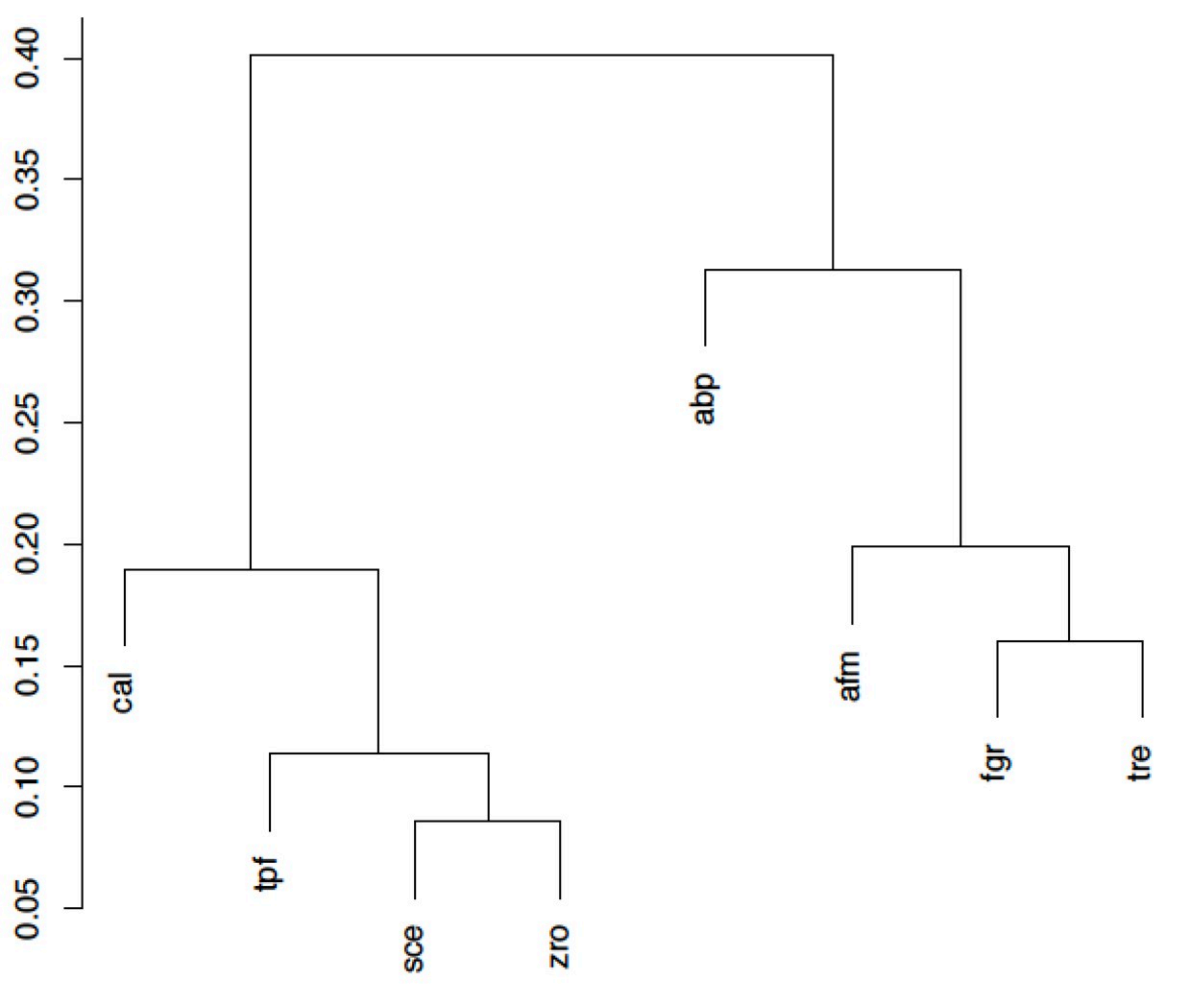
Clustering obtained in the Yeasts and Molds experiment for the pathway similarity index.

After performing all the pairwise comparisons and building the similarity matrices of the global similarity indexes, we are ready to examine the results. Fig 6 shows the dendrogram obtained by applying the complete-linkage hierarchical clustering technique [46] to the similarity matrix of the *pathway similarity index*. We can clearly observe that the index separates at the top level between Yeasts and Molds, as one could expect from a phylogenetic point of view. We obtain the same clustering with all the indexes defined in this paper, even with the structure similarity index: although it uses only the information concerning the shared compounds of the selected organisms, it is able to differentiate the *Saccharomycetes* class.

#### Third experiment: Clustering of Mammals

To reinforce the concept that *MetNet* is of use in the metabolism’s comparative analysis of a set of organisms, we consider the metabolism of all Mammals currently available in the KEGG database and listed in S1 Table.

Once more, we performed the pairwise comparisons of the 66 organisms belonging to the Mammalia class through the command line version of *MetNet* and built the similarity matrices of all the global indexes defined in this paper.

**Fig 7:**
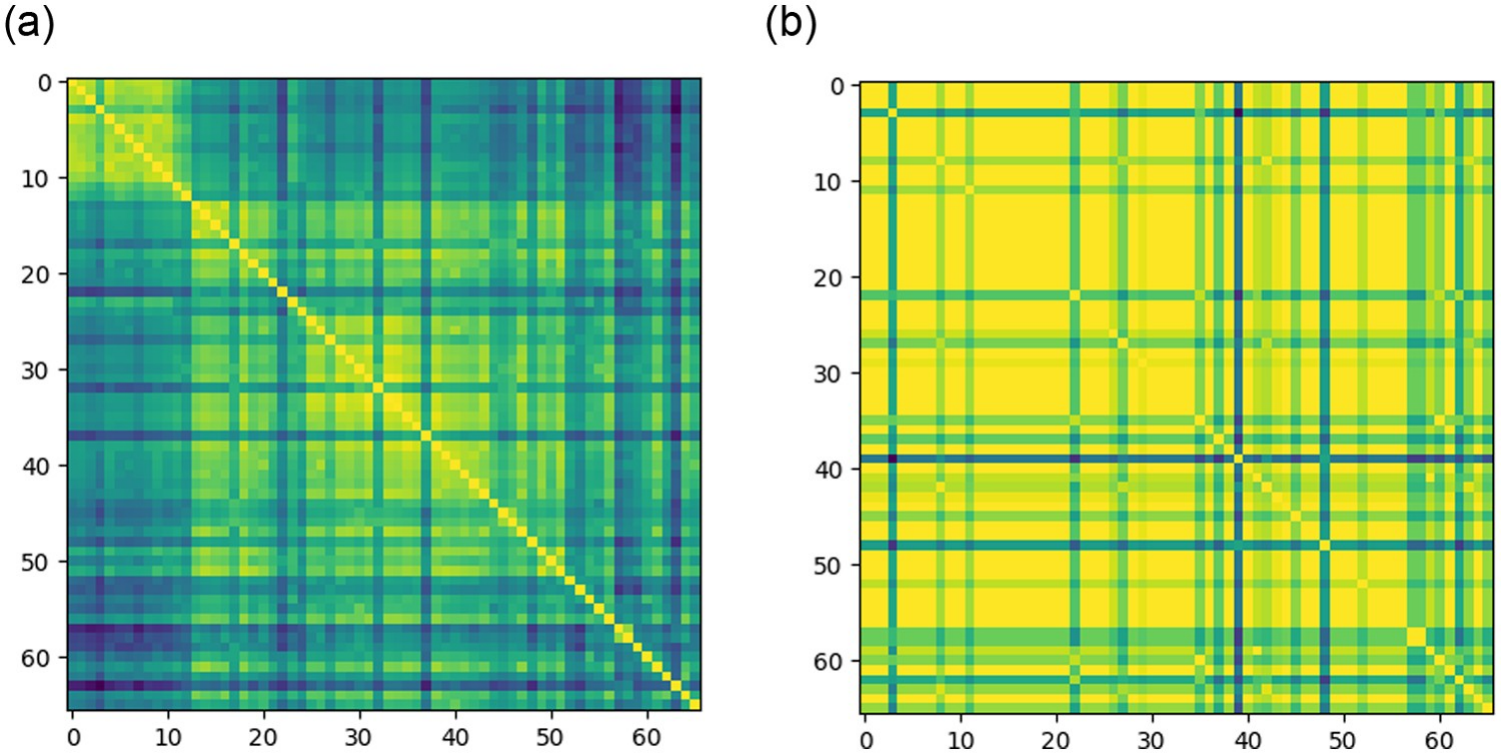
Color map visualization of similarity matrices of the Mammals metabolism experiment: Lighter colors correspond to organisms with high degree of similarity (from yellow to blue). Rows and colums are labeled according to the organisms ids 0–65 listed in S1 Table.

The similarity results between all pairs of considered organisms for the *Weighted pathway similarity index* and the *structure similarity index* can be visually assessed via the similarity matrix rendering reported in Fig 7(a) and 7(b), respectively.

Each depicted similarity matrix has rows and columns labeled according to the organisms ids 0−65 of S1 Table and the cells colors allow for visually comparing the similarity values. In particular, each entry (*i*, *j*) in the matrix shows the similarity value between the *i*-th and *j*-th organisms. The colors range from yellow to blue: yellow corresponds to organisms with the highest degree of similarity while blue corresponds to organisms with the lower degree of similarity. Clearly the main diagonal always shows the yellow color, being the result of the comparison of an organism with itself.

We can easily contemplate that the Weighted pathway similarity index (Fig 7(a)) clearly classifies the organisms 0−12 into the same cluster. This cluster corresponds to all the Primates reported in S1 Table. In addition, we can also observe that the *Structure similarity index* (Fig 7(b) does not separate the Primates into a cluster. This could mean that the metabolic pathways annotated for every mammal are mainly the same, in accordance to the functional and evolutionary entanglement of gene orthology and metabolism [47]. Therefore, the metabolic indexes defined in this work show that the metabolic pathways, in terms of pathway annotations, are the same for all Mammals, but Primates perform the pathway functions differently than any other Mammals.

Finally, although we show here only these two indexes, we remark that all other global indexes defined in the Materials and Methods Section show a similarity matrix similar to the one in Fig 7(a), i.e., they are able to distinguish the Primates.

## Conclusion

In this paper we propose a new approach for reconstructing and comparing the entire metabolism of different organisms, as well as a tool to visualize, explore and measure the obtained results. Our metabolic reconstruction technique is totally automatic and is based uniquely on the KEGG database, which can be a limit wrt. the represented information, allows automatic reconstructions and is a reliable and constantly updated knowledge base.

To manage complexity, our comparison metodology relies on the standardised modularisation of metabolism into reference pathways supplied by KEGG. In fact our metabolism representation is on two levels: the structural level represents relations among metabolic functions (i.e. KEGG reference pathways) and is graph-based; the functional level represents chemical reactions in the corresponding pathway and it is set-based. We introduce similarity indexes to quantify the pairwise comparison of the metabolism of two different organisms. Some indexes measure the local similarity between pathways, at the structural and functional levels separately. Others measure the global similarity between metabolisms, again at the two levels separately. A further index combines together the global structure and pathways similarities into a comprehensive similarity result.

The Java tool *MetNet* implements our proposal. It automatically reconstructs the metabolic network of an organism in KEGG and compares the metabolism between a pair of user selected organisms following the two-level methodology. It provides the quantitative results of the various similarity indexes and offers the possibility to visually explore and compare the metabolic networks.*MetNet* can be used both as an interactive application and as a command line tool. Moreover, thanks to its strong modular structure, the tool can be easily extended with new comparison methods both at a network and at a pathway level.

Some experiments have been performed with *MetNet* in order to validate the proposed methodology. The results we report and discuss are encouraging: the two-level methodology shows to be interesting and effective for metabolism comparisons. Moreover, the visualisation of the metabolic network offered by *MetNet* turns out to be a valuable feature to explore and compare the metabolisms of the organisms under examination. The symbiont-host experiment shows that *MetNet* is a very useful tool to discover and explore metabolic complementations of symbiotic partners in a very small time frame with an intuitive platform, which can then lead to proving relevant metabolic events experimentally with important *a priori* supportive information and specific targets. Also, *MetNet* has proven to be of use when considering the comparison of a set of very similar organisms, like the Yeasts and Molds experiment, as well as in the large scale comparison of all Mammals, being able in both cases to correctly classify them.

As far as future work is concerned, we would like to apply our two-level comparison to metabolic networks reconstructed from experimental genomic data, possibly belonging to more than one organism. This requires the development of an approach to metabolic network reconstruction compliant with the modular organization of the KEGG pathways. Moreover, the tool *MetNet* has to be significantly extended to allow the input of genomic data and implement the reconstruction of the corresponding metabolic network.

## Supporting information

S1 Table List of KEGG’s Mammals. The table reports all mammals considered for the third experiment, identified by a numerical id.
(PDF)
