# Supplementary material for: MetNet: A two-level approach to reconstructing and comparing metabolic networks
Source: PLoS One. 2021 Feb 12;16(2):e0246962. doi: 10.1371/journal.pone.0246962 (PMC7880445; doi:10.1371/journal.pone.0246962)
Supplement: S1 Table — The table reports all mammals considered for the third experiment, identified by a numerical id. (PDF) [file pone.0246962.s001.pdf]

S1 Table: List of KEGG's Mammals

| Id | Code | Organism |
|---|---|---|
| 0 | *hsa* | Homo sapiens (human) |
| 1 | *ptr* | Pan troglodytes (chimpanzee) |
| 2 | *pps* | Pan paniscus (bonobo) |
| 3 | *ggo* | Gorilla gorilla (western lowland gorilla) |
| 4 | *pon* | Pongo abelii (Sumatran orangutan) |
| 5 | *nle* | Nomascus leucogenys (northern white-cheeked gibbon) |
| 6 | *mcc* | Macaca mulatta (rhesus monkey) |
| 7 | *mcf* | Macaca fascicularis (crab-eating macaque) |
| 8 | *csab* | Chlorocebus sabaeus (green monkey) |
| 9 | *rro* | Rhinopithecus roxellana (golden snub-nosed monkey) |
| 10 | *rbb* | Rhinopithecus bieti (black snub-nosed monkey) |
| 11 | *cjc* | Callithrix jacchus (white-tufted-ear marmoset) |
| 12 | *sbq* | Saimiri boliviensis boliviensis (Bolivian squirrel monkey) |
| 13 | *mmu* | Mus musculus (mouse) |
| 14 | *mcal* | Mus caroli (Ryukyu mouse) |
| 15 | *mpah* | Mus pahari (shrew mouse) |
| 16 | *rno* | Rattus norvegicus (rat) |
| 17 | *mun* | Meriones unguiculatus (Mongolian gerbil) |
| 18 | *cge* | Cricetulus griseus (Chinese hamster) |
| 19 | *ngi* | Nannospalax galili (Upper Galilee mountains blind mole rat) |
| 20 | *hgl* | Heterocephalus glaber (naked mole rat) |
| 21 | *ccan* | Castor canadensis (American beaver) |
| 22 | *ocu* | Oryctolagus cuniculus (rabbit) |
| 23 | *tup* | Tupaia chinensis (Chinese tree shrew) |
| 24 | *cfa* | Canis familiaris (dog) |
| 25 | *vvp* | Vulpes vulpes (red fox) |
| 26 | *aml* | Ailuropoda melanoleuca (giant panda) |
| 27 | *umr* | Ursus maritimus (polar bear) |
| 28 | *uah* | Ursus arctos horribilis |
| 29 | *oro* | Odobenus rosmarus divergens (Pacific walrus) |
| 30 | *elk* | Enhydra lutris kenyoni (northern sea otter) |
| 31 | *mdo* | Monodelphis domestica (opossum) |
| 32 | *pcw* | Phascolarctos cinereus (koala) |
| 33 | *fca* | Felis catus (domestic cat) |
| 34 | *ptg* | Panthera tigris altaica (Amur tiger) |
| 35 | *ppad* | Panthera pardus (leopard) |
| 36 | *aju* | Acinonyx jubatus (cheetah) |
| 37 | *bta* | Bos taurus (cow) |
| 38 | *biu* | Bos indicus (zebu cattle) |
| 39 | *bom* | Bos mutus (wild yak) |
| 40 | *bbb* | Bubalus bubalis (water buffalo) |
| 41 | *chx* | Capra hircus (goat) |
| 42 | *oas* | Ovis aries (sheep) |
| 43 | *ssc* | Sus scrofa (pig) |
| 44 | *cfr* | Camelus ferus (Wild Bactrian camel) |
| 45 | *cdk* | Camelus dromedarius (Arabian camel) |
| 46 | *bacu* | Balaenoptera acutorostrata scammoni (minke whale) |
| 47 | *lve* | Lipotes vexillifer (Yangtze River dolphin) |
| 48 | *oor* | Orcinus orca (killer whale) |
| 49 | *dle* | Delphinapterus leucas (beluga whale) |
| 50 | *pcad* | Physeter catodon (sperm whale) |
| 51 | *ecb* | Equus caballus (horse) |
| 52 | *epz* | Equus przewalskii (Przewalski's horse) |
| 53 | *eai* | Equus asinus (ass) |
| 54 | *myb* | Myotis brandtii (Brandt's bat) |
| 55 | *myd* | Myotis davidii |
| 56 | *mna* | Miniopterus natalensis |
| 57 | *hai* | Hipposideros armiger (great roundleaf bat) |
| 58 | *dro* | Desmodus rotundus (common vampire bat) |
| 59 | *pale* | Pteropus alecto (black flying fox) |
| 60 | *ray* | Rousettus aegyptiacus (Egyptian rousette) |
| 61 | *mjv* | Manis javanica (Malayan pangolin) |
| 62 | *lav* | Loxodonta africana (African savanna elephant) |
| 63 | *tmu* | Trichechus manatus latirostris (Florida manatee) |
| 64 | *shr* | Sarcophilus harrisii (Tasmanian devil) |
| 65 | *oaa* | Ornithorhynchus anatinus (platypus) |
